# Collaborative review of pilot projects to inform policy: A methodological remedy for pilotitis?

**DOI:** 10.1186/1743-8462-5-17

**Published:** 2008-07-19

**Authors:** Pim Kuipers, John S Humphreys, John Wakerman, Robert Wells, Judith Jones, Philip Entwistle

**Affiliations:** 1 Centre for Remote Health (a joint Centre of Flinders University & Charles Darwin University), Alice Springs, NT, Australia; 2 Monash University School of Rural Health, Bendigo, Victoria, Australia; 3 Menzies Centre for Health Policy & College of Medicine and Health Sciences, Australian National University, Acton, ACT, Australia

## Abstract

**Background:**

In rural health and other health service development contexts, there is frustration with a reliance on pilot projects as a means of informing policy and service innovation. There is also an emerging recognition that existing research methods do not draw lessons from the failed sustainability that characterises many of these pilots and demonstration projects.

**Discussion:**

This article describes critical aspects of the methodology of a successful collaborative, multi-method, systematic synthesis of exemplary primary health care pilot projects in rural and remote Australia, which synthesised principles from a number of pilot projects to inform policy makers and planners. Hallmarks of the method were: the nature of the source materials for the research, the subsequent research engagement with the actual pilot projects, the extent of collaboration throughout the study with end-users from policy and planning arenas, and the attention to procedural quality.

**Summary:**

The methodology, while time consuming, has resulted in applied, policy-relevant findings, and evidence of consideration by policy-makers.

## Background

Within health service development, 'pilotitis' might be understood as dissatisfaction (of service funding agencies, government departments and service providers) with isolated pilot projects which may have been successful, but were not rolled out into enduring changes in broader service provision or policy. A recent web-search using the term 'pilotitis', reflected this phenomenon. Within the first page of ten 'hits' there were links to transcripts from the UK House of Commons [1], the Scottish Parliament [2], consultations of the Welsh Assembly [3] and the Australian Government Senate Hansard [4]. Of note, all of these related to government level discussions and concerns pertaining to the health service arena. While bordering on the frivolous, this anecdotal observation may also be reflective of a broader discontent across Western governments and departments of health regarding pilot projects, one-off trials and exploratory implementation of demonstration models.

This apparent dissatisfaction expressed by bureaucrats recognises that while disparate trials and pilot interventions are worthwhile, their benefit often only extends to a small group, invariably ceases on completion of the project, and that the lessons learned at times fail to translate beyond the final report to sustainable outcomes [5]. We suggest that this frustration reflects a general feeling within government that there is a need to translate the lessons learned from pilots into key principles, generalisable beyond one or a few pilot projects, to inform policy and health service planning.

Unfortunately, traditional systematic reviews have not been able to fulfil this function. Due to constraints of scope as well as methodological and philosophical limitations, the utility of systematic reviews to inform broader policy or service planning needs, has not yet been realised [6]. In response, this paper outlines aspects of a methodology which may go some way towards meeting these needs. The methodology was successfully employed in a review of pilot projects which emerged during a decade of innovation [7] in rural and remote primary health care service delivery in Australia. As with other systematic syntheses which seek to inform policy [8], our study included the iterative definition of research questions and the ongoing refinement of a search strategy and keywords. It comprised an extensive search across traditional databases for peer reviewed literature, and utilised multiple strategies to identify unpublished documents. The selection process comprised successive rounds of analysing title, abstract, and main body of publications. Published and unpublished documents that met the inclusion criteria were read and thematically analysed by at least two researchers, relevant concepts were categorized, compared, and summarised.

## Discussion

The actual synthesis methodology we utilised is not new [9]. It is consistent with similar studies which seek to inform policy through conducting systematic qualitative reviews or mixed-methods systematic syntheses [10]. While such reviews are potentially more conducive to policy formulation than other forms of research, they do not automatically translate to actual policy [11]. In order to strengthen this link we sought to emphasise a number of key features, which have resulted in the research engaging constructively with rural health policy [12]. These are discussed below.

### 1. The source materials selected

First, we sought to maximise the relevance of our research by choosing information and data that came from actual service delivery pilot projects. We specifically sought to include as source material, information, publications, and evaluation reports arising from exemplary pilot projects, and projects that had progressed beyond the pilot phase to gain some level of sustained funding (Box 1). We selected peer-reviewed and published ('black') literature, as well as searching broadly and through multiple avenues for relevant unpublished ('grey') literature (see Additional file 1). Acknowledging the distinctive characteristics and limitations of these data, the primary criteria for inclusion of documents comprised a balance of 'relevance' and 'quality'. The inclusion of evaluation reports and project level information alongside peer reviewed publications ensured that our focus remained on identifying successful project outcomes. It is important for researchers to make apparent to potential end-users not only the merit of the research process, but also the suitability and relevance of the data selected.

### 2. Engagement with exemplary pilots identified in the literature review

Having identified several core principles and guidelines underpinning successful primary health care models relevant to small rural and remote communities, we then undertook to validate and expand on our findings. This phase involved conducting a total of 52 interviews across six exemplary rural and remote projects that were identified through the synthesis of the literature. This included site visits in geographically diverse areas, discussions with staff, and direct observation of services and their administration. The interviews sought to understand from the perspective of key players, the requirements and processes underpinning the implementation, sustainability and generalisation of the successful pilots. Purposive sampling was undertaken to include a comprehensive range of people associated with each project from different stages of implementation and fulfilling different roles. Face-to-face semi-structured interviews of approximately one hour were undertaken with stakeholders (Additional file 2). Interviews were recorded and transcribed verbatim. Such detailed information from a variety of key individuals ensured that our conclusions were comprehensive and applied.

### 3. Collaboration with end users

Throughout the research we undertook to facilitate translation of findings by actively involving key 'policy-engaged' stakeholders (potentially the end-users of the findings); an interactive model of policy and research [13]. Recognising that health policy is not based only on evidence, but also influenced through social and professional networks and power relationships [14], we conducted our research in close and repeated collaboration with a reference group of experts who were directly or indirectly engaged with rural health policy development and implementation (Additional file 3). This is consistent with calls for greater engagement between researchers and policy makers, at all stages of the research, from question formulation to interpretation of results [15].

In addition to the active involvement of policy experts in our synthesis, we also sought to enhance the up-take of our research through numerous presentations of findings in fora of relevant policy makers [16-19]. Based on the review findings, interviews were conducted with key stakeholders of exemplary pilot projects identified in the review. These explored aspects of the implementation and performance of the identified exemplary pilots.

### 4. Attention to quality

Finally, we were concerned to appropriately maximise the quality of our research and reflect this to our audience. While measures of reliability and validity are essential quality criteria in traditional research, other considerations were also relevant in this project where our primary interest was to engage policy makers. Hence concepts such as credibility, applicability, transferability and trustworthiness realised through the processes of our research were emphasised as meaningful and pertinent criteria for quality [20]. That is, we recognised that confidence in the research findings would arise from ensuring:

• Credibility and trustworthiness, through the use of multiple researchers, multiple sources, through the active participation of policy reference group members in shaping the process of the research, and the accurate reflection of informant perspectives.

• Applicability, through careful attention to the methodological process, and the extent to which we confirmed and grounded our findings in service realities.

• Transferability, through reference group involvement and making clear connections between the data sources (literature and pilots) and concerns of policy makers.

### Other issues

It is noteworthy that an element of the success of this process was the focus of the research funding body, to expressly fund policy-relevant research, within a limited time frame, and in a format that allows for engagement of key policy makers. The mandate of the Australian Primary Health Care Research Institute (APHCRI) is to support innovative primary health care (PHC) research which informs policy and practice using methods that are appropriate to the available data, and relevant to policy-related research questions. Further, APHCRI is committed to facilitating the uptake of evidence in primary health care policy and practice by supporting high levels of engagement with individuals who are directly involved with policy development and service planning. This support enabled our collaborative research team to investigate a key area of health services research, utilising a mixed-methods approach that paid due attention to quality and rigour.

Despite the advantages of such a policy-oriented research approach, the research team also recognised several limitations. The process of repeated consultation with a reference group of policy-engaged persons made the enterprise more time-consuming and also added considerably to the research costs. The inclusion of data on the basis of relevance as well as quality brought with it some methodological shortcomings, requiring considered and well documented decisions.

We selected pilot projects for inclusion as exemplars, on the basis of the existence of published articles or publicly available reports, including any unpublished evaluation reports relating to those pilots. Hence, the initial criterion may have been subject to a publication bias since our research may have over-represented the more celebrated and evidently successful projects, and may have neglected those which had not sought or not had the opportunity to publish information about their pilot.

## Summary

This paper outlines a successful approach that has enabled us to conduct high quality research and determine key policy-relevant principles across numerous pilot projects for the implementation, sustainability and generalization of PHC initiatives in rural and remote areas (Additional file 4).

In order for a compelling case to be put to policy makers beyond the positive outcomes of individual pilot projects, we recommend the adoption of methodologically diverse approaches to systematic review to ensure that as many meaningful and relevant data are included as possible. Furthermore, we suggest that health services researchers should move beyond reviewing the literature, to actually engaging with projects on the ground. This fieldwork provides a more comprehensive and robust understanding and validation of factors associated with pilot projects. We also advocate maximum engagement with policy makers in as many aspects and phases of the research as possible. Such research requires substantial investment and foresight on the part of funding bodies and a willingness to prioritise innovation and relevance alongside more traditional forms of methodological rigour.

## Competing interests

The Australian Primary Health Care Research Institute funded the research projects on which the article is based.

## Authors' contributions

PK and JH conceived of the article and PK led the writing. All authors were actively involved in the research projects and in review and editing of drafts. All authors read and approved the final manuscript.

## Supplementary Material

Additional file 1 Click here for file

Additional file 2 Click here for file

Additional file 3 Click here for file

Additional file 4 Click here for file
