# Supplementary material for: Collaborative review of pilot projects to inform policy: A methodological remedy for pilotitis?
Source: Aust New Zealand Health Policy. 2008 Jul 19;5:17. doi: 10.1186/1743-8462-5-17 (PMC2503987; doi:10.1186/1743-8462-5-17)
Supplement: Additional file 1 [file 1743-8462-5-17-S1.pdf]

- Material was identified through databases of peer reviewed literature as well as a variety of other means
- Where a model was known, but little information could be found in the black literature, additional material was sourced from internet searches using key words relevant to the particular model.
- In consultation with a specialist librarian, we located published papers identified through detailed electronic database searches, including medical and broader health-related databases.
- We adopted a pragmatic approach to the selection of grey literature. We searched through reference lists of sourced materials, search engines via identified keywords, websites of government departments, workforce agencies, professional associations, universities and similar organisations. We also included works known to the researchers, works known to the reference group members, and other research establishments.
- Publications were assessed for quality and relevance, with an emphasis on relevance.

Box 1. Source material for the systematic review
