# Supplementary material for: Collaborative review of pilot projects to inform policy: A methodological remedy for pilotitis?
Source: Aust New Zealand Health Policy. 2008 Jul 19;5:17. doi: 10.1186/1743-8462-5-17 (PMC2503987; doi:10.1186/1743-8462-5-17)
Supplement: Additional file 2 [file 1743-8462-5-17-S2.pdf]

- Interviews were conducted to confirm and elucidate the findings from the systematic review
- Interviews sought a variety of perspectives, including those of:
    - Officers and managers in relevant federal and state government departments involved in implementation and support of project
    - Managers and implementers of each exemplar pilot project
    - Staff employed in the project
    - Associated organisations
    - Consumers
    - Other professional groups.

<u>Box 2. Subsequent interviews with stakeholders associated with exemplary pilot projects and projects which have become sustainable services through mainstream funding</u>.
