# Supplementary material for: Collaborative review of pilot projects to inform policy: A methodological remedy for pilotitis?
Source: Aust New Zealand Health Policy. 2008 Jul 19;5:17. doi: 10.1186/1743-8462-5-17 (PMC2503987; doi:10.1186/1743-8462-5-17)
Supplement: Additional file 3 [file 1743-8462-5-17-S3.pdf]

- Comprised key stakeholders and end-users (policy makers at state and federal government levels, experts in the area)
- Advised on the conceptualisation, focus and scope of the study. Informed the refinement of the research questions. Advised on search terms used. Commented on inclusion / exclusion criteria
- Assisted with the identification of relevant source material (particularly ‘grey’ literature).
- Advised on the policy drivers and the ways in which study findings can be most useful for policy makers.

Box 3. Reference Group involvement
