# Supplementary material for: Collaborative review of pilot projects to inform policy: A methodological remedy for pilotitis?
Source: Aust New Zealand Health Policy. 2008 Jul 19;5:17. doi: 10.1186/1743-8462-5-17 (PMC2503987; doi:10.1186/1743-8462-5-17)
Supplement: Additional file 4 [file 1743-8462-5-17-S4.pdf]

In summary we suggest that:

- Researchers should consider the benefits of drawing on alternative source materials (including evaluation reports) as potential data for systematic reviews of service delivery issues.
- Researchers should also recognise the benefit of direct research engagement with actual pilot projects.
- Policy makers and researchers should recognise the benefits of ongoing collaboration with policy end-users, and make time and resources available accordingly.
- Funders of research should consider the complexity of engaging with the policy arena and implications for the commissioning specific research activity

Such research, combined with due processes for quality may enable the research community to go some way towards addressing the problem of 'pilotitis'.

Box 4: Summary
